# Simulation and team training embedded nurse mentoring programme and improvement in intrapartum and newborn care in a low-resource setting in Bihar, India

**DOI:** 10.7189/jogh.10.021010

**Published:** 2020-12

**Authors:** Rakesh Ghosh, Hilary Spindler, Jessica Dyer, Amelia Christmas, Susanna R Cohen, Aritra Das, Sunil Sonthalia, Tanmay Mahapatra, Aboli Gore, Hemant Shah, Dilys M Walker

**Affiliations:** 1 Global Health Sciences, University of California San Francisco, San Francisco, California, USA; 2 PRONTO International, Seattle, Washington, USA; 3 PRONTO International, Patna, Bihar, India; 4 College of Nursing, University of Utah, Salt Lake City, Utah, USA; 5 CARE India, Bihar, India; 6 Department of Obstetrics and Gynecology and Reproductive Services, University of California San Francisco, San Francisco, California, USA

## Abstract

**Background:**

Improvement of the quality of maternal and child health care remains a focus in India. Working with the Government of Bihar, CARE-India facilitated a comprehensive set of quality of care improvement initiatives. PRONTO’s simulation and team-training was incorporated into the large-scale *Apatkaleen Matritva evam Navjat Tatparta* (AMANAT)nurse-mentoring program of the Government of Bihar supported by CARE-India to improve maternal and child health outcomes. Along-with the AMANAT program, the PRONTO components provided training on nontechnical and technical competencies for managing a variety of obstetric and neonatal conditions, as a team. This study assessed the effectiveness of nurse-mentoring including simulations on intrapartum and newborn care practices in 320 basic emergency obstetric and neonatal care (BEmONC) facilities.

**Methods:**

Deliveries were observed to obtain specific information on evidence-based practice (EBP) indicators before and after the intervention. Intrapartum and newborn care composite scores – were calculated using those EBP indicators. A web-based routine monitoring system provided total training days, weeks and days/week of training and counts of simulation and teamwork-communication activities. Multilevel linear regression was used to examine the exposure-outcome associations.

**Results:**

The final analysis included 668 normal spontaneous vaginal deliveries (NSVDs) from 289 public health facilities in Bihar. Facility-level intrapartum and newborn scores improved by 37 and 26-percentage points, respectively, from baseline to endline. Compared to the bottom one-third facilities that performed fewest NSVD simulations, the top one-third had 6 (95% confidence interval (CI) = 1-12) percentage points higher intrapartum score. Similar comparison using maternal complication simulations yielded 7 (95% CI = 1-12) percentage point higher scores. The highest newborn scores were observed in the middle one-third of facilities relative to the bottom one-third that did the fewest NSVD simulations (5, 95% CI: 1-10).

**Conclusions:**

Findings suggest significant overall improvement in intrapartum and newborn care practices after the AMANAT nurse-mentoring program in public sector BEmONC facilities. Simulation and team-training likely contributed towards the overall improvement, especially for intrapartum care.

**Study registration:**

ClinicalTrials.gov number NCT02726230.

Over the last 15 years, efforts to reduce maternal and neonatal mortality have galvanized globally. Still, a focus on quality of care to reach the Sustainable Developments Goals remains relevant, especially for India where 17% of the global maternal deaths occurred during 2013 [1]. Furthermore, four Indian states together, including Bihar, account for almost 15% of all neonatal deaths globally [2]. Evidence suggests that strengthening of obstetric and neonatal services is imperative to reduce maternal and neonatal mortality [3-5].

Mentoring has been increasingly used to improve quality of maternal and child health services in India [6-8]. Most notably, ‘*Dakshata*’, is one such mentoring program under the National Health Mission, being implemented in seven Indian states [6]. The Government of India’s LaQshya initiative is also expected to improve quality of delivery and immediate newborn care [9]. In Bihar, CARE-India, a non-governmental organization, aiming principally to reduce the mortality and malnutrition among the infants and women of reproductive age, is working with the Government of Bihar since 2011, to facilitate a range of initiatives that evolved into a statewide catalytic process for health system strengthening and improvement of the quality of care. The key components included changes in policies related to human resources and procurements; infrastructure strengthening at facilities; implementation of a model for mentoring and training for clinical care providers; development of functional data systems for procurement and supply chain management of essential drugs and equipment, and program monitoring; functional public-private partnerships related to ambulance, diagnostics, and biomedical waste management services, accreditation and reimbursement guidelines etc. [10]. CARE-India partnered with PRONTO International (a nonprofit organization), University of California, San Francisco (UCSF) and the University of Utah, to integrate highly realistic simulation and team-training into an at-scale nurse-mentoring program called “*Apatkaleen Matritva evam Navjat Tatparta*” (AMANAT), meaning readiness for emergency obstetrical and neonatal care [11-14].

As mentoring programs are implemented across India, it is imperative that these are rigorously evaluated to inform sustainability and scalability efforts. The relative importance of different components of mentoring programs such as skills and drills, didactics, bedside mentoring coupled with simulation and team training are also required to be investigated, in order to better assess the optimal content, dose and duration.

This pre-post comparison study aimed to assess the effectiveness of the customized simulation training, as an add-on component to the larger AMANAT program of the Government of Bihar, on intrapartum and newborn care practices in deliveries that occurred in 320 public Basic Emergency Obstetric and Neonatal Care (BEmONC) facilities at the primary care level in Bihar, India. Specifically, we examined the effectiveness of varying doses of AMANAT nurse-mentoring, performance of simulations, and teamwork-communication activities on normal spontaneous vaginal deliveries (NSVD) that did not develop complications during the time of observations. The SQUIRE 2.0 guidelines to report quality improvement studies were used [15].

## METHODS

### Study setting

The intervention and data collection were conducted between May 2015 and January 2017 in Bihar, India. Bihar has a population of 103 million and is one of the poorest regions in South Asia [12,13]. Health infrastructure in the state is inadequate [14]. In 2015, one BEmONC (primary care) facility served a rural population of approximately 50 000 [16], much higher than the government mandated limit of one facility per 20 000-30 000 population [17], which depicts the stress these facilities are in, constantly. As such, obstetric and pediatric specialists were not available in these facilities and the majority of labor and delivery services were provided by nurses with a two-year Auxiliary Nurse Midwifery qualification completed after secondary education.

### Study subjects

**Figure 1:**
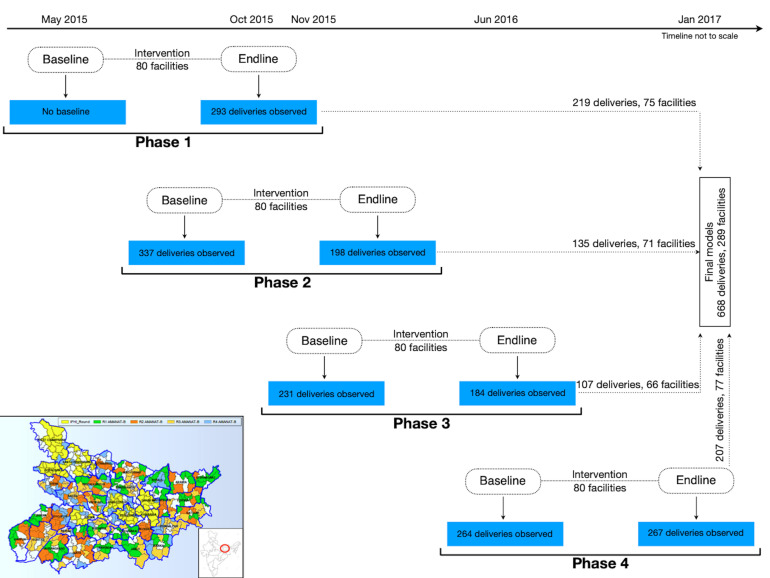
Flowchart of the implementation of the AMANAT program by phases and the geographic distribution of the facilities in the state of Bihar, India.

In total, 1774 deliveries were observed for intrapartum and newborn care practices from the intervened facilities. Of these, 942 were observed post-intervention so that the impact of the intervention on practices could be assessed (**Figure 1**). 179 deliveries developed complications (post-partum hemorrhage, preeclampsia/eclampsia and neonatal complication requiring resuscitation) and were excluded because adequate numbers of observed complications with complete covariate information to analyze by specific outcomes were not available. As we wanted to assess independent practices of mentored nurses only, from the remaining 763 deliveries, 34 were excluded where the nurse conducting the direct observation of delivery (DOD) got involved in the clinical management and 61 were excluded due to missing covariate information, leaving 668 NSVDs from 289 facilities for the final analysis.

### Study intervention

During 2011-2013, CARE-India collaborated with the Government of Bihar and other non-governmental organizations to support the Government of Bihar implement a package of reproductive, maternal, newborn and child health and nutritional interventions in eight districts. Expansion of the program to all 38 districts in the state began in 2013, with the overarching goal of improving maternal, newborn and child health [18]. PRONTO’s simulation and team training was integrated into this package of interventions to increase the overall efficiency of the AMANAT program. The program aimed to improve the quality of care in public Emergency Obstetric and Neonatal Care facilities in Bihar by building the capacity of the service providers through in-job, on-site training targeting the improvement of their knowledge and skill, behavior and efficient engagement in other aspects like procurement of supplies.

The program was implemented over four phases. In each phase, pairs of Nurse Mentors were assigned four BEmONC facilities where they conducted on-site mentoring. Each month, Nurse Mentor pairs visited a facility for one week, then moved on to the next until the pair covered all four assigned facilities. They returned for week-long visits every month for eight months [19]. Efforts were made to train all delivery care nurses from each facility and attendance in the training sessions were more than 80% as reported elsewhere [20].

The program facilitated the training for the management of the uncomplicated and complicated deliveries, teamwork and structured communication between providers, and prioritized compassionate and respectful communication with the mother [11,19,21]. The PRONTO curriculum^TM^ was tailored to the unique needs pertaining to the public facilities in Bihar and included simulations in which providers, without adequate prior exposure to clinical scenarios, had to simultaneously manage both the mother and the newborn, in a clinical setting where they work. Simulations involved a maternal actor wearing PartoPants^TM^, a hybrid low-tech birth simulator [11], and a NeoNatalie® infant mannequin. During each site visit, Nurse Mentors were required to conduct simulations of three clinical scenarios involving vaginal deliveries: 1) NSVD, 2) post-partum hemorrhage, and neonatal resuscitation. All Nurse Mentors were trained to run and video record simulations, facilitate post-simulation video-guided debriefing, and conduct post-live birth debriefing. The simulation and team training activities provided mentees with opportunities to practice nontechnical and technical competencies required to manage a variety of obstetric and neonatal situations, as a team.

### Measurements

We used data from the DOD and the Facility Information System (FIS), which were CARE-India’s concurrent assessment platform for the Government of Bihar’s AMANAT program. Clinical enumerators visited each facility for a week to observe deliveries occurring between 9 am and 5 pm. Deliveries were included if all three stages of labor were observed by an independent observer, not from admission to discharge. DOD provided comprehensive information on specific evidence-based practice (EBP) indicators for individual deliveries during the *pre- and post-intervention* periods. FIS was a web-monitoring system gathering data on: patient demographics, delivery information, diagnosis and management of maternal and neonatal complications before and after delivery, along with the discharge or referral information while the *intervention was ongoing*. FIS also provided training details including date, time and topics covered for each activity, number and type of each simulation conducted and staff attendance for each session. It is likely that the same providers were observed at baseline and endline. However, the data set did not have the ability to match baseline with endline delivery observations by providers.

#### Outcome

A panel of experts including obstetricians and gynecologists, neonatologists, midwives and curriculum experts identified a set of EBP indicators recommended by WHO, [22,23] which were assessed during DOD and were also aligned with the mentoring curriculum. Intrapartum and newborn care composite scores, the two outcomes for this analysis, were calculated using the EBP indicators from DOD (see Appendix S1 and Figure S1 in the **Online Supplementary Document**).

#### Exposure

The four exposures used in this analysis were obtained from the FIS data: (1) total training days, (2) the number of training weeks and days per training week, (3) counts of simulations by types and (4) teamwork-communication activities. This information was digitally maintained by Nurse Mentors in their daily training logs using FIS. Additionally, UCSF team independently maintained a record of the dates of site visits along with the number and type of each simulation run in each facility, which was used to validate the simulation counts obtained from FIS.

#### Covariates

We used DOD and FIS data to obtain both maternal and facility level covariate information. Maternal age, caste, gravida, parity, time and type of delivery (NSVD or complicated) were obtained from DOD. As FIS collected information for more days than DOD, we estimated the delivery load per facility per day using the former to obtain more stable estimates.

### Statistical analysis

Individual EBP indicators were assigned a score of 1 or 0 based on whether they were performed as recommended or not, respectively. Given the significance of timely administration of uterotonics to prevent postpartum hemorrhage [24], the expert panel recommended “uterotonics administered in the third stage of labor” be given twice the weight compared to other individual indicators. The indicator-specific scores were aggregated to obtain a total score for each delivery, which was rescaled by dividing with the maximum possible score. The final scores ranged between 0 and 100, where a 0 refers to none and 100 refers to all EBPs being performed appropriately for a delivery. Scores for all deliveries within a facility were averaged to obtain facility-level aggregate scores. Identical indicators were used to generate baseline and endline scores.

Total numbers of training days per facility were obtained from the training dates. Training weeks and days/week were combined to create a four-level training dose variable: “incomplete frequency + dose” = <7 weeks and <5 days per week, “incomplete frequency” = <7 weeks and ≥5 days/week, “incomplete dose” = ≥7 weeks and <5 days/week, and “complete frequency + dose” = ≥7 weeks and ≥5 days/week. Based on clinical content, simulations were categorized into three non-mutually exclusive groups: (1) simulations containing an uncomplicated normal vaginal birth scenario were identified as NSVD simulations, (2) simulations containing any maternal complications were identified as maternal simulations, and (3) simulations with neonatal complications were identified as neonatal simulations (See statistical analysis section in the **Online Supplementary Document**).

As births were clustered within facilities, a multilevel linear regression model with a random intercept for facility was used to examine the exposure-outcome associations. We generated a directed acyclic graph [25] with all available variables to carefully consider their role as potential confounders or mediators before building the statistical model (see Figure S2 in the **Online Supplementary Document**). Change-in-estimate method was used, with at least 5% change in the exposure-outcome association for a variable to qualify to be in the model [26]. We investigated interaction between simulation and teamwork-communication activities using a product term in the model to examine if the joint association of the two was more than the sum of the individual associations. Two-tailed t-test at the 5% level was used for statistical significance. Because of skewed continuous distributions of the counts of simulations and teamwork-communication activities, we additionally analyzed them in categories (tertiles). To determine the effectiveness of training, the endline practice scores were used as outcomes after accounting for the baseline scores of facilities. Baseline data for Phase 1 were not collected, hence those were imputed by averaging the baseline scores for Phases 2 through 4, which were comparable. Phase 1 Nurse Mentors were involved for Phase 3 mentoring and better performing Nurse Mentors of Phases 2 and 3 were engaged for Phase 4. Therefore, we used a fixed effect for phase to model this and other differences across phases. Using the FIS data, we estimated the inverse of the probability that a delivery was selected for observation because of the sampling design and adjusted the models with the inverse probability weights. We examined if the models fulfilled regression assumptions and whether extreme observations influenced the results. For sensitivity analyses, the models were built without excluding deliveries where the clinical enumerator was involved in delivery management. We also fit co-adjusted models with both exposures (ie, simulations and teamwork-communication). Analyses were conducted in STATA 14.0 (Stata Corp, College Station, TX, USA) [27].

### Ethical considerations

This study is part of the *Ananya* Bihar programme and is registered at ClinicalTrials.gov number NCT02726230. The Institutional Review Board of the Indian Institute of Health Management Research (IIHMR) in Jaipur and the Committee for Human Research at UCSF reviewed and approved the study (Approval# 14-15446).

### Role of the funding source

The funding agency had no role in the conception, design and conduct of this assessment or in the analysis and interpretation of the results.

## RESULTS

Of the total 942 deliveries observed at endline, 668 were included in the final analysis, as described earlier. About 70% of all deliveries occurred between noon and 5 pm (**Table 1**). Almost four-fifths of those who delivered in the BEmONC facilities belonged to the socio-economically deprived “other backward” caste, “scheduled caste” or “tribe”. Less than one-third were primipara and mean age at delivery was 24 (standard deviation, SD = 4) years. On average, there were 5 (SD = 3) births per day in the BEmONC facilities during the training period.

**Table 1:** Characteristics of directly observed deliveries from the AMANAT intervention in Bihar, India 2015-2017 (n = 668)*

Characteristics	Percentage (No.) or mean (standard deviation)†
**Phase (%):**	
1	32.8 (219)
2	20.2 (135)
3	16.0 (107)
4	31.0 (207)
**Time of delivery (%):**	
9 am-12 noon	30.5 (204)
12 noon-5pm	69.5 (464)
**Caste (%):**	
Scheduled castes or tribes	31.4 (210)
Other backward castes	50.0 (334)
General	18.6 (124)
**Parity (%):**	
Primipara	27.1 (181)
1	30.1 (201)
2	19.9 (133)
3+	22.9 (153)
**Maternal age (years):**	
Mean (standard deviation)	24.4 (3.5)
Min-max	12-40
**Facility delivery volume (births per day)‡:**	
Mean (standard deviation)	4.7 (2.5)
Min-max	1-17

*Some of the totals may be a little less than 942 because of missing responses.

†Proportions (n) are presented for categorical variables and mean (standard deviation) with the range is presented for the continuous ones.

‡Values are at the facility level and includes all births per day during the mentoring days obtained from FIS.

The mean duration of training was 39 (SD = 5) days across 289 facilities (**Table 2**). About 9% of the facilities received the “incomplete frequency + dose”, 8% received the “incomplete frequency”, 39% received the “incomplete dose” and 44% received the “complete frequency + dose” of training. The mean number of NSVDs, maternal and neonatal complication simulations run per facility across all phases were 24 (SD = 13), 20 (SD = 10) and 13 (SD = 7), respectively. On average, 7 (SD = 6) teamwork-communication activities were conducted per facility.

**Table 2:** Characteristics (mean and standard deviation, or n and %) of PRONTO simulation training at the facilities by phases of the AMANAT intervention in Bihar, India 2015- 2017

	Phase 1 (n = 75)	Phase 2 (n = 71)	Phase 3 (n = 66)	Phase 4 (n = 77)	Overall (n = 289)
Training days (mean count):					
Number of training days	38.1 (3.6)	43.0 (4.1)	38.7 (5.5)	36.7 (3.7)	39.1 (4.9)
Training dosage (%):					
Incomplete frequency + dose*	15.0 (11)	1.4 (1)	9.1 (6)	11.7 (9)	9.3 (27)
Incomplete frequency†	12.0 (9)	0 (0)	10.6 (7)	9.1 (7)	8.0 (23)
Incomplete dose‡	34.7 (26)	49.3 (35)	25.8 (17)	44.2 (34)	38.8 (112)
Complete frequency + dose§	38.7 (29)	49.3 (35)	54.6 (36)	35.1 (27)	43.9 (127)
Simulation type (mean count):					
NSVD simulations‖	18.1 (6.3)	35.8 (14.7)	23.2 (8.3)	20.7 (11.1)	24.3 (12.6)
Maternal simulations¶	15.1 (6.0)	28.4 (11.4)	19.1 (7.5)	17.3 (9.2)	19.9 (10.1)
Neonatal simulations**	10.2 (3.9)	18.9 (8.6)	13.5 (6.1)	11.6 (6.0)	13.4 (7.2)
Teamwork-communication (mean count):					
Teamwork-communication activities	5.8 (3.9)	12.2 (7.2)	5.2 (5.1)	5.3 (4.6)	7.1 (6.1)

*Less than 7 weeks of training and less than 5 d/week, percentage of total facilities within each category.

†Weeks <7 and days/week ≥5.

‡Weeks ≥7 and days/week <5.

§Weeks ≥7 and days/week ≥5.

‖Simulates deliveries with normal spontaneous vaginal deliveries with or without complications.

¶Simulated deliveries with maternal complications.

**Simulated deliveries with neonatal complications.

### Intrapartum care

**Figure 2:**
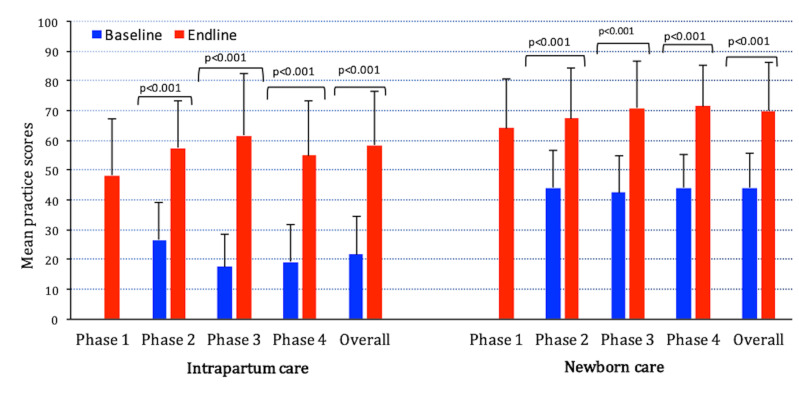
Change in intrapartum and newborn care practice scores (aggregated at the facility level) from baseline to endline by phases of the AMANAT intervention in Bihar, India 2015-2017, n = 203 facilities from DOD. *P-value from the paired *t* test, n for phases 1 through 4 and overall are 75, 69, 62, 72 and 203, respectively. The scores range from 0-100, with 0 referring to none and 100 referring to all of the evidence based practices being performed appropriately. Note: Phase 1 baseline was not conducted. Error bars represent standard deviation.

Overall, intrapartum care composite scores improved by 37 percentage points (95% CI = 34-40) from baseline to endline (**Figure 2**) with significant improvement in individual indicators (see Figure S1 in the **Online Supplementary Document**, Panel 1).

Ten days of training was associated with 6 (95% CI = 1-11) percentage points improvement in intrapartum scores (Table 3), ie, 40 days of training (one week × eighth months ≈ mean training days) was associated with 24 percentage points improvement, about 65% of the overall improvement. Relative to facilities receiving the “incomplete frequency + dose”, those receiving “incomplete frequency”, “incomplete dose” and “complete frequency + dose” of training had 4 (95% CI = -7 to 15), 8 (95% CI = 0.1-16) and 9 (95% CI = 1-17) percentage point higher scores, respectively (P-trend = 0.04, **Table 3**).

**Table 3:** Associations (linear regression coefficients, 95% confidence interval, CI) of characteristics of PRONTO simulation training with intrapartum and newborn care practice scores estimated from direct observation of non-complicated deliveries in the AMANAT intervention in Bihar, India 2015-2017

			Intrapartum care (n_1_)		Newborn care (n_2_)	
	**n_1_/n_2_**	**Exposure increment**	**Percentage points (95% CI)**	***P*-value‖**	**Percentage points (95% CI)**	***P*-value‖**
Training days (continuous)	668/668	Per 10 d	5.9 (0.5, 11.4)	0.03	3.3 (-0.6, 7.2)	0.09
Training dosage:						
Incomplete frequency + dose*	68/68	1	Reference		Reference	
Incomplete frequency†	53/53	2 vs 1	4.4 (-6.6, 15.4)		7.6 (-0.4, 15.5)	
Incomplete dose‡	255/255	3 vs 1	8.3 (0.1, 16.4)		6.0 (0.2, 11.9)	
Complete frequency + dose§	292/292	4 vs 1	8.7 (0.6, 16.8)	0.04^§^	5.5 (-0.4, 11.4)	0.22^§^
NSVD simulations (continuous)¶	621/621	Per 10 sims	1.5 (-0.4, 3.5)	0.12	0.5 (-1.0, 1.9)	0.54
Tertile 1 (4-17)	226/226	1	Reference		Reference	
Tertile 2 (18-26)	204/204	2 vs 1	2.8 (-2.9, 8.5)		5.4 (1.1, 9.6)	
Tertile 3 (27-79)	191/191	3 vs 1	6.3 (0.5, 12.1)	0.03§	4.3 (0.02, 8.6)	0.05§
Maternal/Neonatal simulations (continuous)**	621/621	Per 10 sims	1.7 (-0.7, 4.1)	0.16	0.9 (-1.6, 3.5)	0.48
Tertile 1 (2-15)/(0-10)	245/256	1	Reference		Reference	
Tertile 2 (16-22)/(11-15)	194/185	2 vs 1	2.9 (-3.0, 8.7)		5.3 (1.0, 9.6)	
Tertile 3 (23-60)/(16-43)	182/180	3 vs 1	6.7 (1.0, 12.4)	0.02§	2.3 (-1.9, 6.6)	0.22§
Teamwork-communication activities (continuous)	668/668	Per 10 activities	2.1 (-2.1, 6.3)	0.32	-1.0 (-4.0, 2.0)	0.52
Tertile 1 (0-4 activities)	278/278	1	Reference		Reference	
Tertile 2 (5-9 activities)	205/205	2 vs 1	6.3 (0.8, 11.8)		0.2 (-3.8, 4.2)	
Tertile 3 (10-27 activities)	185/185	3 vs 1	6.4 (0.4, 12.3)	0.02§	1.5 (-2.9, 5.9)	0.53§

*Less than 7 weeks of training and less than 5 d/week, percentage of total facilities within each category.

†Weeks <7 and days/week ≥5.

‡Weeks ≥7 and days/week <5.

§Weeks ≥7 and days/week ≥5.

‖*P*-value for the t-test of linear trend across ordered categories. All models are adjusted for facility level baseline scores, phase of intervention, total facility delivery volume, time of delivery, age and caste of the mother, parity of the index pregnancy, inverse probability weights and restricted to normal deliveries where mentees alone handled the deliveries without any mentor involvement.

¶Simulates deliveries with normal spontaneous vaginal deliveries with or without complications.

**Intrapartum care was examined with simulations that had maternal complications and newborn care with neonatal complication simulations. The range within parenthesis for each category refers to the number of sims that constitute the category.

Relative to the lowest one-third of the facilities where fewest NSVD simulations were conducted, those in tertiles two and three had 3 (95% CI = -3 to 9) and 6 (95% CI = 1-12) percentage point higher scores, respectively (P-trend = 0.03, **Table 3**), which is 8% and 16% of the overall improvement. Associations with maternal complication simulations were similar to NSVD simulations and accounted for about 18% of the overall improvement. Significant associations were also observed with teamwork-communication activities; facilities that performed five to nine and 10 to 27 teamwork-communication activities had 6 (95% CI = 1-12) and 6 (95% CI = 0.4-12) percentage point higher intrapartum scores, respectively, compared to the facilities that performed zero to four activities (P-trend = 0.02, **Table 3**). None of the above associations were significant on a continuous scale (**Table 3**).

### Newborn care

Newborn care scores improved by 26 percentage points (95% CI = 24-29) from baseline to endline (**Figure 2**) with improvement in individual indicators, except for those where baseline adherence was almost 90% (see Figure S1 in the **Online Supplementary Document**, Panel 2).

Training duration was not significantly associated with newborn scores. Facilities with “incomplete dose” of training had a significant, 6 percentage point (95% CI = 0.2-12) increase in newborn care scores compared to facilities that had the “incomplete frequency + dose” of training, with no dose-response trend (P-trend = 0.2, **Table 3**).

The number of NSVD simulations run was significantly associated with newborn scores only for the middle one-third of the facilities (tertile two) with 5 percentage point (95% CI = 1-10) higher newborn scores compared to facilities that did fewest NSVD simulations (P-trend = 0.05). Similarly, performing neonatal simulations significantly improved newborn scores only for the middle one-third of the facilities (tertile two) relative to facilities that did the fewest neonatal simulations, with no dose-response trend (P-trend = 0.2). There was no association between teamwork-communication activities and newborn scores (**Table 3**).

In sensitivity analyses, the same models as above were built after including cases where the clinical enumerator was involved. The inclusion marginally attenuated the magnitude and the strength of the associations (see Table S1 in the **Online Supplementary Document**). There was no interaction between simulations and teamwork-communication for any of the two scores.

## DISCUSSION

Findings suggest facility-level intrapartum and newborn care practices improved significantly from pre to post intervention, for NSVDs in BEmONC facilities in Bihar. About 65% of the overall improvement in intrapartum scores from baseline to endline could be ascribed to successful completion of the entire training. Performing NSVD or maternal complication simulations, as a part of the training, was associated with about 8%-18% of the overall improvement in intrapartum scores. A similar proportion of the overall improvement in intrapartum score was also observed to be associated with the performance of teamwork-communication activities. As there was no significant interaction, the combined improvement from simulation and teamwork-communication activities appeared to be potentially additive [28]. Therefore, simulation and team-training together probably accounted for about one-third of the overall change in intrapartum scores from baseline to endline (results from co-adjusted models support this conclusion, see Table S2 in the **Online Supplementary Document**).

Intrapartum scores showed a dose-response trend with training dosage; more completed weeks of training were associated with relatively greater magnitude of improvement than fewer and incomplete weeks of training, although the marginal difference may not be clinically significant. For newborn scores, training of longer duration did not appear to accrue value beyond a point. Newborn care related topics were covered early in the training; therefore, for newborn care it was beneficial if early weeks were complete. Having more weeks later in the training, when the early weeks were incomplete, did not appear to compensate for the missed time, probably because competing curricular topics were to be covered each week. Disproportionate weightage resulting from relatively fewer simulations on newborn topics in the curriculum could also be a potential explanation. Smaller magnitude of association between number of training days with newborn score (3 percentage points) compared to intrapartum score (6 percentage points) supports this explanation (**Table 3**).

NSVD simulations or maternal simulations appeared to have a dose-response relationship with intrapartum score. A significant association was observed only among the set of facilities that performed the highest numbers of simulations (tertile three). For newborn score, the results suggest that performing simulations less than a threshold number could not bring a significant improvement, beyond which there is no additional benefit.

Several programs in low- and middle-income countries are aimed at improving quality of maternal and child health services. In India, the *Dakshata* program supported by Jhpiego, implements mentorship and use of a standardized case sheet [6]. The Helping Babies Survive (HBS) and Helping Mothers Survive (HMS) programs, which provide low-cost, low-tech drill-based jobsite training spaced over time [7,29], has some similarity to the AMANAT program facilitated by the PRONTO simulation and team-training component. Both programs resulted in improved use of EBPs. For example, both HBS/HMS and AMANAT appeared to improve handwashing by 23% and 48%. respectively. Respective values were 18% vs 51% for uterotonic administration and 28% vs 35% for skin to skin contact (reference [7] and Figure S1 in the **Online Supplementary Document**). Given the complexity of factors influencing providers’ clinical behavior, the diversity of settings where the programs were implemented and lack of substantiation by clinical outcome data, cautious interpretation of these comparisons is warranted. Instilling EBPs are intermediate steps in achieving improved clinical outcomes. Of note, the recent Better Birth Trial demonstrated that using the WHO Safe Childbirth Checklist improved practices [30], but resulted in no obvious significant improvement in maternal and child health outcomes [31].

The study had some limitations. Exposure and outcome information were gathered using two different systems of data collected, by different groups of clinical enumerators. While the exposure data was collected during the intervention, outcomes were assessed before and after the intervention. These aspects generated some potential for information bias, which is likely to be non-differential, as the clinical enumerators conducting endline DOD were unaware of the training details for their facilities. In other words, chances of biased assessment of practices in favor of facilities with more training or simulations, and vice-versa, was very low. Nurses, while being observed, could have adhered to EBPs more as opposed to when unobserved [32]. Thus, Hawthorne effect was a possibility, though it should be present in both base and endline, potentially nullifying each other. As there were no facilities without intervention, the analysis lacked direct controls for comparison. Pre–post longitudinal comparison within facilities over time is a robust design tantamount to a time-stratified case-crossover comparison, where facilities served as their own controls, minimizing facility level time-independent confounding. Nevertheless, there may be some time-varying residual confounding. All deliveries observed occurred between 9 am and 5 pm. It is possible that the observed practices may not be reflective of the practices outside these hours. To address this limitation, we used FIS data that captured all births to estimate the inverse of the probability that a birth was observed because of the study design and adjusted the models with the inverse probability weights. This adjustment should account for potential differential deviations from EBPs during deliveries beyond the observation hours as opposed to the observed hours. Additionally, only a few deliveries were observed per facility, which may not be truly reflective of the actual practices of the nurses in the facilities. Furthermore, simulation training was an add-on component under the bigger umbrella of AMANAT intervention and other quality improvement efforts. The statistical models that showed associations with simulations and teamwork-communication activities were adjusted for a range of potential confounders. However, the actual impact on practices could be due to a host of factors (including the role of other components of AMANAT) not accounted for in the models. Teasing out specific impact and attributing to simulation was impossible because of the integrated nature of the intervention. Hence the interpretations of the impact-related findings were made for the overall program. Additionally, for Phase 1 as there were no actual baseline, data were imputed, as described earlier. The baseline status for Phase 1 facilities could actually have been different from others as they were selected to be mentored first because they had higher level of preparedness than others.

## CONCLUSIONS

This study provides evidence of overall improvement in facility-level intrapartum and newborn care practices in NSVDs as an impact of the AMANAT intervention. Simulations and teamwork-communication activities likely contributed to the overall improvement, especially for intrapartum care. This study generates empirical evidence to the global audience on the effectiveness of simulation and team training delivered through a nurse-mentoring program, which is a useful strategy to instill the use of EBPs during live births among nurses working at BEmONC facilities in low-resource settings.

## Additional material

Online Supplementary Document
