# Supplementary material for: Simulation and team training embedded nurse mentoring programme and improvement in intrapartum and newborn care in a low-resource setting in Bihar, India
Source: J Glob Health. 2020 Dec 19;10(2):021010. doi: 10.7189/jogh.10.021010 (PMC7759018; doi:10.7189/jogh.10.021010)
Supplement: Online Supplementary Document [file jogh-10-021010-s001.pdf]

ONLINE SUPPLEMENT

Appendix S1.

Outcome

The intrapartum score was comprised of 11 EBPs: (1) checked blood pressure before delivery, (2) checked pulse before delivery, (3) monitored fetal heart sound in first stage of labor, (4) monitored fetal heart sound in second stage of labor, (5) checked for cord around fetus's neck, (6) uterotonics administered in the third stage of labor, (7) controlled cord traction performed, (8) uterus massaged, (9) checked for completeness of placenta and (10) checked for completeness of membranes and (11) delivery attendant wore gloves. The newborn score was comprised of 12 EBPs: (1) infant placed on mother's abdomen immediately after delivery, (2) cord pulsation checked before clamping, (3) cord clamped after waiting for one-two minutes, (4) sterile cord clamps used, (5) cord was cut using sterile blade, (6) skin-to-skin contact initiated, (7) eyes wiped with sterile gauze, (8) newborn dried, (9) newborn wrapped in clean dry cloth, (10) cord stump was left clean, (11) weight taken at birth and (12) infant was breast fed within an hour after birth.

Statistical analysis

The training dates for each facility were aggregated to obtain the total number of training days. We combined the number of training weeks and days per training week to create a four-level categorical variable and defined "incomplete frequency + dose" – <7 weeks and <5 days per week, "incomplete frequency" – <7 weeks and ≥5 days/week, "incomplete dose" – ≥7 weeks and <5 days/week and "complete frequency + dose" – ≥7 weeks and ≥5 days/week. Based on clinical content, simulations were identified into three non-mutually

exclusive groups groups; (1) simulations containing a birth scenario with or without complications were identified as NSVD simulations, (2) simulations containing any maternal complications with or without newborn complications were identified as maternal simulations, and (3) simulations with neonatal complications with or without maternal complications were identified as neonatal simulations.

As a second component of the PRONTO curriculum, we used the total number of T&C activities performed in each facility during the training period. Simulations and T&C activities were analyzed as both continuous and categorical exposures. The distributions of the simulations and the T&C activities were positively skewed. To minimize an undue influence of extreme values on the effect estimates, the exposures were categorized into tertiles. Furthermore, tertiles also helped in exploring any non-linear relationships such as a threshold effect.

Supplement Figure 1. Change in intrapartum (panel 1) and newborn (panel 2) care in the direct observation of non-complicated delivery indicators from baseline to endline by phases of the AMANAT intervention in Bihar, India 2015 - 2017.

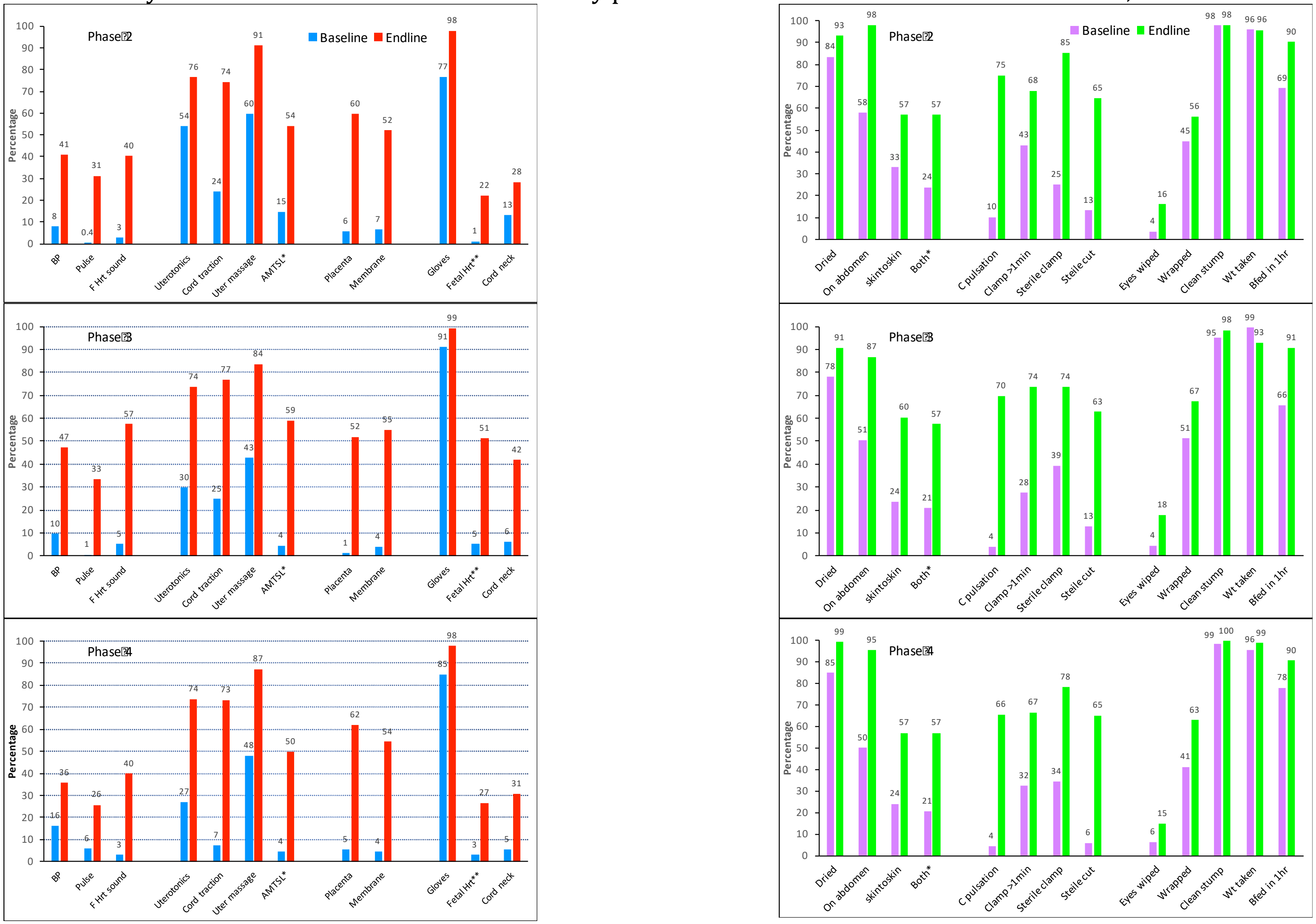

* Intrapartum care – all three steps of AMTSL were performed, newborn care – both means baby was placed on abdomen immediately after delivery and skin-to-skin care was given.

** Fetal heart sound was monitored in the second stage of labor.

Supplement Figure 2. Conceptual framework showing the relationship of PRONTO training with change in intrapartum and newborn care practice scores between baseline and endline. It shows the potential confounders that might affect this relationship.

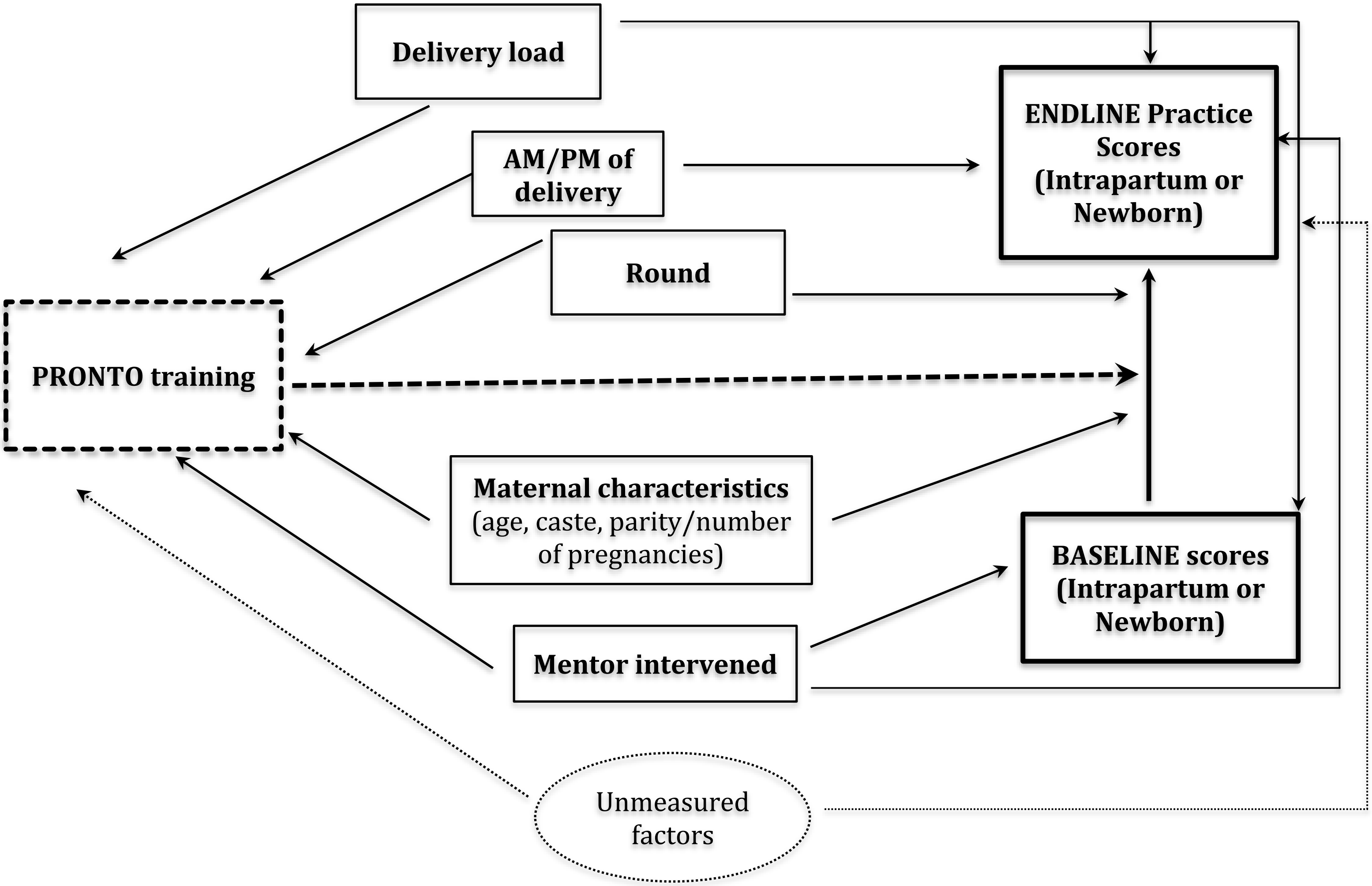

Supplement Table 1. Associations (linear regression coefficients, 95% Confidence Interval, CI) of characteristics of PRONTO simulation training with intrapartum and newborn care practice scores estimated from direct observation of *all*† non-complicated deliveries in the AMANAT intervention in Bihar, India 2015 - 2017.

			**Intrapartum care (n_1)**		**Newborn care (n_2)**	
	n_1/n_2	Exposure increment	Percentage points (95% CI) ‡	P-value	Percentage points (95% CI) ‡	P-value
Training days (continuous)	700/700	Per 10 days	5.3 (-0.2, 10.7)	0.06	3.3 (-0.6, 7.2)	0.10
Training dosage						
Incomplete frequency + dose *	68/68	1	Reference		Reference	
Incomplete frequency **	53/53	2 vs. 1	4.2 (-6.9, 15.3)		7.5 (-0.5, 15.5)	
Incomplete dose ***	274/274	3 vs. 1	8.2 (0.9, 16.4)		5.9 (0.03, 11.8)	
Complete frequency + dose ****	305/305	4 vs. 1	8.3 (0.2, 16.5)	0.06§	5.3 (-0.6, 11.2)	0.24§
NSVD simulations (continuous)†	648/648	Per 10 sims	1.5 (-0.4, 3.4)	0.12	0.5 (-0.9, 1.9)	0.49
Tertile 1 (4 – 17 sims)	238/238	1	Reference		Reference	
Tertile 2 (18 – 26 sims)	207/207	2 vs. 1	2.5 (-3.3, 8.2)		5.2 (1.0, 9.5)	
Tertile 3 (27 – 79 sims)	203/203	3 vs. 1	5.6 (-0.2, 11.4)	0.06§	4.1 (-0.2, 8.4)	0.06§
Maternal/Neonatal simulations (continuous)#	648/648	Per 10 sims	1.7 (-0.7, 4.0)	0.17	1.0 (-1.6, 3.5)	0.47
Tertile 1 (2 – 15) / (0 – 10)	256/262	1	Reference		Reference	
Tertile 2 (16 – 22) / (11 – 15)	198/194	2 vs. 1	2.3 (-3.6, 8.1)		5.1 (0.9, 9.4)	
Tertile 3 (23 – 60) / (16 – 43)	194/192	3 vs. 1	5.9 (0.2, 11.6)	0.04§	2.2 (-2.0, 6.5)	0.24§
Teamwork-communication activities (continuous)	700/700	Per 10 activities	1.9 (-2.2, 6.0)	0.37	-0.7 (-3.7, 2.3)	0.65

Tertile 1 (0 – 4 activities)	296/296	1	Reference		Reference	
Tertile 2 (5 – 9 activities)	210/210	2 vs. 1	5.9 (0.4, 11.3)		-0.02 (-4.0, 4.0)	
Tertile 3 (10 – 27 activities)	194/194	3 vs. 1	6.3 (0.4, 12.3)	0.02§	1.8 (-2.6, 6.2)	0.46§

† All directly observed deliveries without excluding those where the clinical enumerator was involved in assisting the provider.
‡ Adjusted for facility level baseline scores, phase of intervention, total facility delivery volume, time of delivery, age and caste of the mother, parity of the index pregnancy, inverse probability weights and restricted to normal deliveries.
* Less than 7 weeks of training and less than 5 days/week, percentage of total facilities within each category,
** Weeks <7 and days/week ≥5,
*** Weeks ≥7 and days/week <5,
****Weeks ≥7 and days/week ≥5.
§ P-value for the chi-square test of linear trend across ordered categories.
† Simulates deliveries with normal spontaneous vaginal deliveries with or without complications.
\# Intrapartum care practice was examined against simulated deliveries with maternal complications and newborn care against neonatal complication related simulations.

Supplement Table 2. Associations (linear regression coefficients, 95% Confidence Interval, CI) of simulation and teamwork and communication activities with intrapartum and newborn care practice scores from simulation and teamwork and communication co-adjusted models.

		Simulation		Teamwork-Communication	
	Exposure increment	Percentage points (95% CI) ‡	P-value	Percentage points (95% CI) ‡	P-value
**Intrapartum care**					
NSVD simulations†					
Tertile 1 (4 – 17)	1	Reference		Reference	
Tertile 2 (18 – 26)	2 vs. 1	3.3 (-2.4, 9.0)		6.6 (1.0, 12.2)	
Tertile 3 (27 – 79)	3 vs. 1	5.2 (-0.7, 11.1)	0.10§	5.7 (-0.4, 11.8)	0.04§
Maternal simulations#					
Tertile 1 (2 – 15)	1	Reference		Reference	
Tertile 2 (16 – 22)	2 vs. 1	3.1 (-2.6, 8.8)		6.2 (0.6, 11.8)	
Tertile 3 (23 – 60)	3 vs. 1	5.4 (-0.4, 11.1)	0.06§	5.7 (-0.3, 11.8)	0.04§
**Newborn care**					
NSVD simulations†					
Tertile 1 (4 – 17)	1	Reference		Reference	
Tertile 2 (18 – 26)	2 vs. 1	5.4 (1.1, 9.7)		0.6 (-3.6, 4.8)	
Tertile 3 (27 – 79)	3 vs. 1	4.1 (-0.3, 8.6)	0.06§	1.0 (-3.6, 5.6)	0.87§
Neonatal simulations$					
Tertile 1 (0 – 10)	1	Reference		Reference	
Tertile 2 (11 – 15)	2 vs. 1	5.2 (1.0, 9.5)		0.5 (-3.7, 4.7)	
Tertile 3 (16 – 43)	3 vs. 1	2.0 (-2.3, 6.4)	0.27§	1.2 (-3.4, 5.8)	0.77§

‡ Adjusted for facility level baseline scores, phase of intervention, total facility delivery volume, time of delivery, age and caste of the mother, parity of the index pregnancy, inverse probability weights, restricted to normal deliveries, simulation and teamwork and communication activities.
§ P-value for the chi-square test of linear trend across ordered categories.
† Simulates deliveries with normal spontaneous vaginal deliveries with or without complications.
# Simulated deliveries with maternal complications.
$ Simulated deliveries with neonatal complications.
